# Pollution Level and Health Risk Assessment of PM_2.5_-Bound Metals in Baoding City Before and After the Heating Period

**DOI:** 10.3390/ijerph15102286

**Published:** 2018-10-18

**Authors:** Yixuan Liu, Shanshan Li, Chunyuan Sun, Mengxi Qi, Xue Yu, Wenji Zhao, Xiaoxiu Li

**Affiliations:** 1 Beijing Key Laboratory of Resources Environment and Geographic Information System, Capital Normal University, Beijing 100048, China; 2160902137@cnu.edu.cn (Y.L.); 2140902114@cnu.edu.cn (C.S.); 2160902114@cnu.edu.cn (M.Q.); yx150972@163.com (X.Y.); Lxiaoxiu0548@sina.com (X.L.); 2 Base of the State Key Laboratory of Urban Environmental Process and Digital Modelling, Beijing 100048, China; 3 Beijing Municipal Research Institute of Environmental Protection, Beijing 100037, China; liss0502@163.com; 4 Monitoring Center of Beijing Water Environment, Beijing 100038, China

**Keywords:** PM_2.5_, heavy metal, pollution level, health risk assessment

## Abstract

In order to assess the pollution levels and health risks of PM_2.5_-bound metals in Baoding City before and after the heating period, samples were collected in 2016 at Hebei University from September 25th to November 14th during the non-heating period, and November 15th to December 26th during the heating period, respectively. ICP-MS was applied to analyze seven heavy metals (Cr, Zn, Cu, Pb, Ni, Cd and Fe). The statistical analysis, enrichment factor (EF), pollution load index method, and Risk Assessment Method proposed by U.S. EPA were used to evaluate the non-carcinogenic risks of six of these heavy metals (Cr, Zn, Cu, Pb, Ni and Cd) and carcinogenic risks of three of these heavy metals (Cr, Ni and Cd). The results showed three main results. First, the average daily PM_2.5_ concentrations of the national air monitoring stations was 155.66 μg·m^−3^ which was 2.08 times as high as that of the second level criterion in China (75 μg·m^−3^) during the observation period. Compared with the non-heating period, all heavy metals concentrations increased during heating period. The growth rates of Pb and Ni were the highest and the lowest, which were 88.03 and 5.11 percent, respectively. Second, the results of enrichment factor indicated that the EF values of all heavy metals were higher during the heating period in comparison with during the non-heating period, but the degree of enrichment of all heavy metals remained unchanged. Not only those, Cr and Ni were minimally enriched and were affected by both human and natural factors, Pb, Cu and Zn were significantly enriched and were mainly affected by human factors, the enrichment of Cd was much higher than that of the other heavy metals, exhibiting extremely high enrichment, mainly due to human factors during the whole sampling period. The results of the pollution load index indicated that the proportions of the number of highly and very highly polluted PM_2.5_-bound metals were the highest during the heating period, while the proportion of moderately polluted PM_2.5_-bound metals was the highest during the non-heating period. The combined pollution degree of heavy metals was more serious during the heating period. Third, according to the health risk assessment model, we concluded that the non-carcinogenic and carcinogenic risks caused by inhalation exposure were the highest and by dermal exposure were the lowest for all kinds of people. The overall non-carcinogenic risk of heavy metals via inhalation and subsequent ingestion exposure caused significant harm to children during the non-heating and the heating periods, and the risk values were 2.64, 4.47, 1.20 and 1.47, respectively. Pb and Cr exhibited the biggest contributions to the non-carcinogenic risk. All the above non-carcinogenic risks exceeded the standard limits suggested by EPA (HI or HQ < 1). The carcinogenic risk via inhalation exposure to children, adult men and women were 2.10 × 10^−4^, 1.80 × 10^−4^, and 1.03 × 10^−4^ during the non-heating period, respectively, and 2.52 × 10^−4^, 2.16 × 10^−4^ and 1.23 × 10^−4^ during the heating period, respectively. All the above carcinogenic risks exceeded the threshold ranges (10^−6^~10^−4^), and Cr posed a carcinogenic risk to all people.

## 1. Introduction

In recent years, with economic development and industrial expansion, long-term and persistent air pollution incidents have come to occur frequently, and complex regional atmospheric pollution problems have also become increasingly significant [1,2,3]. Air pollution is particularly prominent in the Yangtze River Delta, Pearl River Delta, Guanzhong area, and other urban economic belts, especially Beijing-Tianjin-Hebei region [4,5]. With environmental governance on the agenda, the issue of atmospheric pollution has attracted increasing attention from the public, with a particular focus on fine particulate matter (PM_2.5_).

When PM_2.5_ (aerodynamic diameter ≤ 2.5 μm) is one of the air pollutants, these particles can not only reduce visibility but also have a severe negative impact on climate change in global and regional areas [6,7,8]. Moreover, the surface of this particulate fraction could be used as the carrier of viruses, bacteria and heavy metals. Some of these particles will enter the body through the respiratory system, increasing morbidity and mortality due to cardiovascular diseases [9,10]. Not only that, a recent Italian study shows that living in towns at various levels of air pollution during childhood will play an important role in the development of chronic diseases in adulthood, including cancer [11]. Heavy metals, as significant chemical components in PM_2.5_, come from either natural sources or human sources like industrial production (e.g., As, Ag, Cd, Cr, Cu, Mn, Zn, Fe, and Pb), residential heating (e.g., As, Hg, K, Pb, and Rb), automobile exhaust emissions (Sb, Cr, Ba, Mn, Co, Fe, Cu, and Pb) and so on [12,13,14,15]. Heavy metals associated with the PM_2.5_ fraction may enter the human body through dermal, inhalation, and ingestion pathways [16,17]. Studies have shown that a certain amount of Cd in the body is not harmful and ingested Pb can mostly be eliminated by human excretion [18,19,20]. Although the human body has a certain tolerance and clearance mechanism for heavy metals, a great deal of research shows that high levels of heavy metals seriously affect human health. For example, Cr can cause respiratory problems like cough, asthma and bronchitis [21,22]; Cu can damage organs in the body, leading to cirrhosis and other diseases [23]; Cd can damage the kidney, liver and gastrointestinal tract, causing cancer [24,25]; Zn can lead to anemia, poisoning and other symptoms [26]; and prolonged exposure to concentrations of Pb can cause neuropathy [27]. Additionally, the U.S. Environmental Protection Agency has identified Cr, Ni and Cd to be carcinogenic substances [28]. Therefore, it is important to design preventive measures based on the evidence-based medicine, evaluate the health risks and establish risk alarms according to their pollution levels [29].

At present, the research on PM_2.5_ in China and abroad mainly focuses on heavy metals and their sources. For example, Yu et al. [30] analyzed the seasonal variation characteristics and the sources of PM_2.5_ in 2010 and found that the contributions of combustion sources and dust were the highest in the spring and winter, and the contributions of automobile exhaust and industrial production did not show any seasonality. Tao et al. [31] analyzed the seasonal variation characteristics of carcinogenic heavy metals in PM_2.5_ in April, July and October of 2009 and January of 2010 in Beijing and found the enrichment factors of As, Cd, Pb and Se to be significantly higher in summer. The four heavy metals in summer came mainly from regional transmission of pollution sources, while the sources of As and Pb, Se and Cd were mainly due to combustion of coal, urban motor vehicle emissions, and industrial production around Beijing in spring and autumn, respectively. Chen et al. [3] analyzed the sources of heavy metals in PM_2.5_ from October to December in 2012 in Tianjin and discovered that the main sources of heavy metals were coal combustion, chemical pollution and automobile exhaust emissions. Also, there are multiple studies on the health risks of toxic PM_2.5_-bound metals [32,33], and scholars in related fields at home and abroad have mainly focused on the research of direct-controlled municipalities and provincial capitals. For example, Hu et al. [34] evaluated the heavy metals health risks in PM_2.5_ from May to October in 2012 and found that the risks of Cd and Cr were much higher than those of Cu, Pb and Zn, and all five heavy metals posed the highest risk to men and the least to children. Hu et al. [35] analyzed the health risks of Cu, Mn, Co, Cd, Zn, Cr, Ni and Pb in Nanjing in 2010 and found that potential non-carcinogenic risks caused by inhalation exposure were greater than those caused by dermal and ingestion exposure. Chen et al. [3] evaluated the health risks of Mn, Cu, As, Cr, Zn, Cd, Hg, Pb and Ni, found that nine heavy metals could cause significant risks to humans through inhalation exposure. Gao et al. [36] evaluated the heavy metals health risks in PM_2.5_ in seven functional areas in October of 2013 in Beijing and found that Pb posed the highest non-carcinogenic risk in rural areas and inner suburbs, and that the Cr risk level in residential areas, schools and parks exceeded the human acceptance limit. Though these results comes from different exposure scenarios, these studies above show that PM_2.5_-bound metals will pose health risks to all population age groups in many metropolises, to some extent. Therefore, more and more attention should be paid the health risks caused by PM_2.5_-bound metals in small cities. Nowadays, with the economic integration of the Beijing-Tianjin-Hebei pattern, Baoding plays an increasingly important role in the regional economy coordinated development of the Beijing-Tianjin-Hebei region, but there are few studies on the pollution levels and health risks of PM_2.5_-bound metals in Baoding. Hence, it is important to evaluate the pollution levels and health risks of PM_2.5_-bound metals before and after the heating period in Baoding.

In this research, a sampling point was set up at the university in Baoding to collect PM_2.5_ samples from 25 September to 26 December 2016. The pollution levels and health risks of PM_2.5_-bound metals were analyzed in order to provide references for atmospheric prevention and warning in Baoding.

## 2. Materials and Methods

### 2.1. Sample Collection

**Figure 1:**
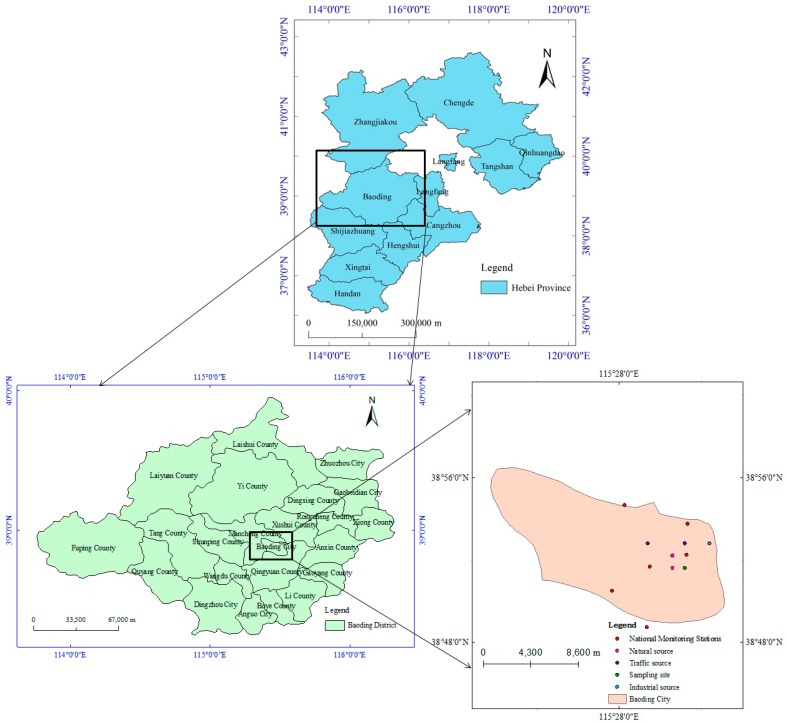
Location of sampling site of Baoding city, China.

As shown in Figure 1, the sampling point was located on the roof (40 m height) of the library at Hebei University (38.88° N, 115.52° E), which is surrounded by residential areas. There was no industrial source of pollutants within two kilometers, so this sampling point was assumed to be representative of the typical environment of Baoding City. PM_2.5_ samples were gathered from September 25th to December 26th in 2016, and daily average concentrations of PM_2.5_ were collected from the national air monitoring stations simultaneously. The non-heating period was from September 25th to November 14th, and the heating period was from November 15th to December 26th. During the sampling period (starting at 9:00 a.m. each day and ending at 8:00 a.m. next day), PM_2.5_ samples were gathered by a TH-150A α intelligent volume sampling instrument (1.13 L·min^−1^; Wuhan Tianhong Intelligent Instrument Factory, Wuhan, China) with 90 mm quartz fiber filters (Whatman, Maidstone, UK), and the machine’s flow recorder can compute each total volume automatically. The sampling instrument was up to EPA standards. Meteorological parameters such as wind speed, wind direction, temperature, and humidity were also recorded at the time of sample collection. Sampling was stopped when it rained or the equipment failed. Not only that, blank samples were treated in the same manner as exposed samples except PM_2.5_ collection for a blank control group. During the non-heating period, there were 19 samples that were discarded, five of which due to wet filters, eight of which due to equipment failure and six due to weather conditions. During the heating period, there were voided 14 samples, four of which were due to wet filters, six due to equipment failure and four due to weather condition. Therefore, there were 32 and 27 samples during the non-heating period and heating period in total, respectively.

### 2.2. Sample Analysis

Before sampling, the filter membranes were prepared according to the sampling technology requirements for environmental air particulates. The filter membranes were balanced in a temperature and humidity-controlled chamber for 24 h and were weighed with a high precision of 10 μg electronic balance (TB-215D, -Denver Instrument, Denver, CO, USA) in a clean room before and after sampling.

Each sample was cut into quarters. Then all samples were treated by microwave digestion and placed in a 100 mL polyethylene digestion tank. All the samples were digested in an 8 mL mixture containing 6 mL of HNO_3_, 2 mL of HCl and 0.2 mL of HF using MARS (CEM, Matthews, NC, USA) for 3 h. After cooling down to room temperature, the digested solution was filtered into 100 mL Teflon vessels and then quantitatively transferred into 100 mL Teflon flasks and refilled with distilled water to 100 mL volume. In the experiments, the digestion solutions were used in the blank test and the blank filter membranes were used in the controlled trial [37].

According to previous studies, the health risks assessments of Cd, Pb, Cu, Ni, Cr and Zn in PM_2.5_ were increasing widely because of the chronic non-carcinogenic risks of these heavy metals [19]. Cd, Cr and Ni had carcinogenic risks [13]. Therefore, it was reasonable to assess the health risks of these six heavy metals in Baoding city. So in this research, inductively coupled plasma mass spectrometry (ICP-MS, 7500a, Agilent, Palo Alto, CA, USA) was applied to analyze seven elements (Cd, Ni, Cr, Cu, Zn, Pb and Fe) of the samples in Baoding. The standard metal solutions of 0.5, 2, 10, and 50 ppb were mixed with 5% nitric acid solution. The mass concentration of each element was measured by an internal standard method. The concentrations of metals in the blank filter membranes were determined following the sample analysis process, and were subtracted from the metals analysis results. To ensure the accuracy of the instrument analysis results, the national marine sediment primary standard substances (GBW07316, GBW07315) and the U.S. Geological Survey basalt standard substances (BHVO-2, BCR-2) were applied for quality control, and the relative standard deviation of all measured elements were less than 10%. The standard recovery for all heavy metals ranged from 86.3% to 103.6%. The definite values was 86.3–95.6%, 94.5–101.3%, 97.2–103.6%, 95.4–102.3%, 96.3–102.4%, 97.2–102.7% and 89.5–98.1% for Cr, Ni, Cu, Cd, Zn, Pb and Fe, respectively.

### 2.3. Pollution Assessment Methods

#### 2.3.1. Enrichment Factor

The Enrichment Factor (*EF*) is a significant target of the level of the interference to the natural environment generated by human activities. *EF* is used to assess the impact of human activities on the natural environment, and calculated by the equation [38]:

(1) $$EF=\frac{{\left(C_{m}/C_{n}\right)}_{\mathrm{environment}}}{{\left(C_{m}/C_{n}\right)}_{\mathrm{soil}\mathrm{crust}}}$$

Here, *C_m_* stands for heavy metals concentrations, and *C_n_* stands for the reference element concentration. The selection of reference elements should meet certain criteria, such as having stable chemical properties which are less influenced by human activity. Hence, Fe is chosen to be the reference element [39]. (*C_m_*/*C_n_*)_environment_ describes as the concentration ratio of heavy metal to the reference element; (*C_m_*/*C_n_*)_soil crust_ describes as the concentration ratio of the corresponding element in the soil to the reference element. In Baoding, the background values of each element in the soil were derived from the China National Environmental Monitoring Center [40]. If *EF* is greater than 10, we can conclude that the element is mainly affected by human activities; if *EF* is less than 1, it can be referred that the element is mostly affected by natural factors; if *EF* is between 1 and 10, the element can be regarded as originating from a complicated combination of natural and artificial factors. Based on *EF* values, the enrichment degree and source are divide into 5 levels [41], listed in Table 1.

#### 2.3.2. Pollution Load Index

Pollution load index is comprised of the pollution from a variety of heavy metals, and reflects the pollution degree of all heavy metals in a sampling point, directly [42]. The calculation formula [43] is as follows:

(2) PLI=∏i=1nCiBin

Here, *PLI* stands for the pollution load index of the elements, *C_i_* stands for the measured concentration of heavy metal and *B_i_* represents the background value of that metal in local soil. According to the pollution load index, pollution is divided into six levels [44,45], as shown in Table 2.

#### 2.3.3. Human Health Risk Assessment Method

In this research, six heavy metals were examined. Cd, Cr and Ni are carcinogenic elements [46]. Pb, Cu and Zn are non-carcinogenic elements [47]. The carcinogenic risks posed by three heavy metals (Cd, Cr and Ni) and the non-carcinogenic risks posed by six heavy metals (Cr, Pb, Ni, Cd, Cu and Zn) to the public were analyzed in this study. In this study, “human population” was divided into three groups: children, adult males and adult females. The public is mainly exposed to metals through the following pathways: dermal contact absorption, inhalation, and direct ingestion [48,49]. The dose of the three pathways can be calculated by the following equation [50]:

CD_Idermal_ = (c × SA × AF × ABS × EF ×ED × CF)/(BW × AT), (3)

CD_Iinhale_ = (c × IR_i_ × EF × ED × CF)/(BW × AT), (4)

CD_Iingest_ = (c × IngR × EF × ED × CF)/(BW × AT), (5)

where C (ng·m^−3^) stands for exposure concentration in PM_2.5_. The variable CD (mg·(kg·d)^−1^) is the dose contacted through dermal contact absorption (CD_Idermal_), inhalation (CD_Iinhale_) and ingestion (CD_Iingest_). Exposure parameters are mainly based on the exposure parameter manual of the population in China [51], as shown in Table 3.

The assessment of non-carcinogenic risks uses the following equation [50]:

(6) $$\mathrm{HI}=\sum_{i=1}HQ_{i}=\sum_{i=1}\left(CDI_{i}/RfD_{i}\right),$$

Here, *HQ* stands for the non-carcinogenic risk of a given pathway, *RfD* (mg·(kg·d)^−1^) stands for the reference dose that can result in non-carcinogenic risk, and *i* stands for the pathway [52,53]. HI represents the non-carcinogenic risk as a result of multiple pathways. Theoretically, when HI or HQ is less than 1, there is no significant non-carcinogenic risk. The *RfD* values of every heavy metal by three exposure pathways are shown in Table 4 [50,54]:

The carcinogenic risks were assessed as follows [51]:

(7) $$R=CDI_{i}*SF_{i},$$

Here, R is the carcinogenic risk, and *SF* (mg·(kg·d)^−1^) stands for the reference dose that can cause carcinogenic risk. In theory, when the acceptable value is between 10^−6^ and 10^−4^, there is no significant health hazard [55]. The *SF* values of carcinogenic heavy metals via three exposure pathways are shown in Table 5 [50,54]:

## 3. Results and Discussion

### 3.1. Concentrations of Heavy Metals

During the whole sampling period, the average daily PM_2.5_ concentrations of the national air monitoring stations was 155.66 μg·m^−3^ which was 2.08 times as high as that of the second level criterion in China (75 μg·m^−3^) [56]. The average daily PM_2.5_ concentrations before and after the heating period all exceeded the standard and were 120.94 and 190.39 μg·m^−3^, respectively. During the non-heating period, the 24 h metals concentrations was 23.24–80.65, 101.18–947.46, 4.49–35.50, 5.41–44.68, 32.09–643.71 and 112.02–853.36 ng·m^−3^ for Cr, Ni, Cu, Cd, Zn, Pb and Fe, respectively. And the average concentrations of Cr, Pb, Ni, Cd, Cu and Zn were 45.04 ± 15.35, 402.11 ± 341.38, 11.93 ± 3.31, 18.83 ± 13.16, 165.41 ± 150.47, and 441.23 ± 236.12 ng·m^−3^, respectively. During the heating period, the 24 h metals concentrations was 29.70–88.90, 220.44–1230.81, 6.28–35.50, 10.14–47.12, 35.61–737.05 and 115.72–1030.66 ng·m^−3^ for Cr, Ni, Cu, Cd, Zn, Pb and Fe, respectively. And the average concentrations of Cr, Pb, Ni, Cd, Cu and Zn were 54.98 ± 17.86, 756.07 ± 409.90, 12.54 ± 5.57, 20.34 ± 8.57, 210.80 ± 191.19 and 547.08 ± 352.52 ng·m^−3^, respectively. Average PM_2.5_-bound metal contents in Baoding during the sampling period are depicted in Table 6. Compared with the non-heating period, all heavy metals concentrations increased during heating period. The growth rates of heavy metals from the highest to the lowest were below: Pb, Cu, Zn, Cr, Cd and Ni, and the growth rates of concentrations of Pb and Ni were 88.03 and 5.11 percent, respectively. PM_2.5_-bound metals concentrations of the representative cities in domestic studies are summarized in the following paragraphs.

Compared to Beijing [31], all heavy metals concentrations in Baoding were higher than those found in Beijing, and the concentrations of Cd and Ni in Baoding were 7.51 and 3.05 times higher than those found in Beijing, which represented the highest and lowest metal concentrations, respectively. Compared to Tianjin [57], all heavy metal concentrations in addition to Zn were higher in Baoding. The concentrations of Cd and Ni were 19.52 and 2.16 times as high as those found in Tianjin, which represented the highest and lowest metal concentrations, respectively. All heavy metals concentrations were higher compared to those found in Chengdu [58], with Cd and Pb being 3.31 and 1.76 times higher than those found in Chengdu, which represented the highest and the lowest metal concentrations, respectively. All heavy metals concentrations in Baoding were higher than those found in Shanghai [59], and the concentrations of Cd and Zn were the highest and lowest, measured at 11.48 and 1.42 times the values found in Shanghai. Compared to Guangzhou [60], all heavy metals in addition to Pb were lower in Baoding than those measured in Guangzhou. Compared to Chongqing [61], all heavy metals in addition to Pb and Cu were lower in Baoding than those measured in Chongqing. All heavy metals concentrations in Baoding were higher than those measured in Xiamen [60], especially Cd, which was 10.84 times higher than the Cd measured in Xiamen. All PM_2.5_-bound metals were high, with Cd concentrations being the highest.

### 3.2. Pollution Evaluation

#### 3.2.1. Analysis of Enrichment Factors

**Figure 2:**
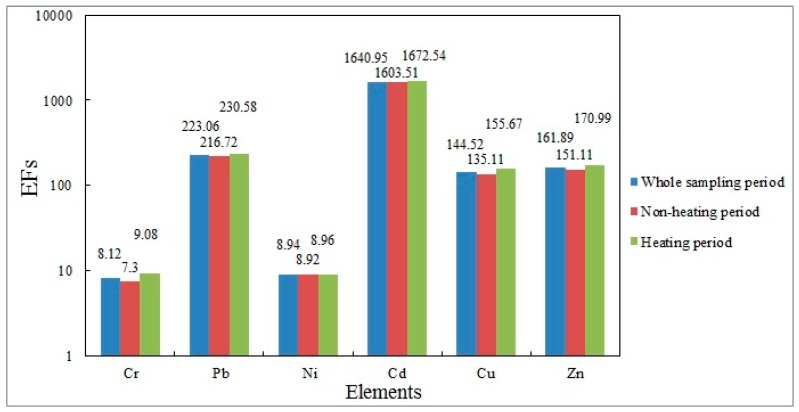
The *EF* values of heavy metals.

The results of the enrichment factor analysis are depicted in Figure 2, the *EF* values for Cr, Pb, Ni, Cd, Cu and Zn were 8.12, 223.06, 8.94, 1640.95, 144.52 and 161.89, respectively, during the whole sampling period, demonstrating that all heavy metals were affected by human factors to varying degrees. Among them, the *EF* values of Cr and Ni belonged to Grade 2, indicating that Cr and Ni were mildly enriched and affected by natural sources and human sources together. The *EF* values for Pb, Cu and Zn belonged to Grade 4, indicating significant enrichment, the *EF* value of Cd belonged to Grade 5, indicating extremely high enrichment, and these values meant that Pb, Cu, Zn, and Cd were affected mainly by human factors.

During the non-heating period, the *EF* values for heavy metals in descending order were Cd (1603.51), Pb (216.72), Zn (151.11), Cu (135.11), Ni (8.92) and Cr (7.30), and the degree of enrichment of each heavy metal were consistent with the observation period. During the heating period, the *EF* values for heavy metals in descending order were Cd (1672.54), Pb (230.58), Zn (170.99), Cu (155.67), Cr (9.08) and Ni (8.96). Compared with the non-heating period, the *EF* values for all heavy metals were increased to different degrees, but the degree of enrichment of all elements remained unchanged.

According to the *EF* values during the non-heating and heating periods, the results showed that the concentration difference of each heavy metal was not obvious. However, the *EF* values for Cd were much higher than those of other heavy metals and the content of Cd was higher than that of other heavy metals, consistent with the conclusion in the previous Section 2.1.

#### 3.2.2. Analysis of Pollution Load Index

**Figure 3:**
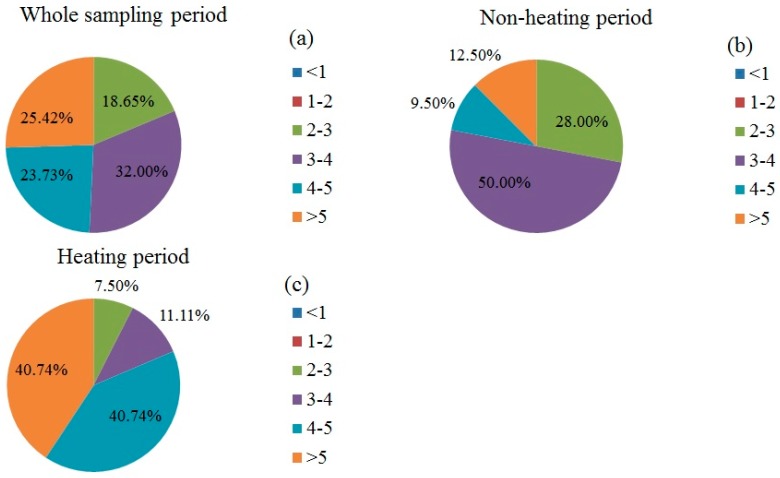
The distribution frequency of the pollution load index in different ranges.

The pollution load index (*PLI*) method was applied to analyze the combined pollution level of all heavy metals in Baoding, and the results are shown in Figure 3. The distribution frequency of *PLI* during the whole sampling period is depicted in Figure 3a. After calculation, the daily *PLI* at the sampling point was between 2.36 to 8.37, indicative of daily levels at the sampling point between Grade 3 and Grade 6, and showing that the area was polluted every day to different degrees. The percentages of lightly pollution, moderately pollution, significantly pollution and severely pollution of PM_2.5_-bound metals were 18.65%, 32.00%, 23.73% and 25.42%, and the percentage of moderately pollution and lightly pollution of PM_2.5_-bound metals were the highest and lowest, respectively.

The results during the non-heating period are shown in Figure 3b, with the daily *PLI* at the sampling point falling between 2.36 to 7.52. This represented a daily level between Grade 3 and Grade 6, indicating that the area was polluted every day to different degrees. The percentages of lightly pollution, moderately pollution, significantly pollution and severely pollution of PM_2.5_-bound metals were 28.00%, 50.00%, 9.50% and 12.50%, respectively, with moderately pollution and significantly pollution being the highest and lowest, respectively. The results during the non-heating period are shown in Figure 3c, with the daily *PLI* at the sampling point falling between 2.62 and 8.37. The percentages of lightly pollution, moderately pollution, significantly pollution and severely pollution of PM_2.5_-bound were 7.50%, 11.11%, 40.74% and 40.74%, respectively, indicating that significantly pollution and severely pollution were most common and lightly pollution were least common. In comparison with the non-heating period, the proportion of lightly polluted and moderately polluted PM_2.5_-bound metals decreased significantly, and the proportion of significantly polluted and severely polluted PM_2.5_-bound metals increased dramatically, indicating that the pollution of heavy metals were more serious during the heating period compared with during the non-heating period.

### 3.3. Health Risk Assessment

#### 3.3.1. Non-Carcinogenic Risk

##### Non-Carcinogenic Risks through the Dermal Exposure Pathway

In Baoding, the non-carcinogenic risks of heavy metals through dermal exposure were analyzed during the whole sampling period and the results are shown in Table 7. The health risks for all of the studied heavy metals during the non-heating period were highest for children, followed by adult females and then adult males. For all population age groups and all sex groups, the non-carcinogenic risks decreased following the trend Pb > Cd > Cr > Cu > Zn > Ni. The non-carcinogenic risks for each heavy metal and the total non-carcinogenic risk were less than 1, indicating that the overall non-carcinogenic risk due to heavy metals was low and was with the acceptable range for the dermal exposure pathway. The non-carcinogenic risks among the metals during the heating period showed the same trend as that observed during non-heating period. In comparison with the non-heating period, the non-carcinogenic risks for all heavy metals increased, but the non-carcinogenic risks and the total non-carcinogenic risks were still less than 1, meaning that the non-carcinogenic risk for heavy metals through the dermal exposure pathway was low. Therefore, the non-carcinogenic risk for heavy metals via the dermal exposure pathway was low during the whole sampling period, and all categories of people were most affected by Pb.

##### Non-Carcinogenic Risks through the Inhalation Exposure Pathway

For different populations, the non-carcinogenic risks of heavy metals via the inhalation exposure pathway during the whole sampling period are depicted in Table 8. The non-carcinogenic risks via inhalation were the highest to children, followed by adult males and then adult females during the non-heating period. For all three population groups, the non-carcinogenic risks via inhalation descended following the trend Pb > Cd > Cr > Cu > Zn > Ni. The total heavy metal-related non-carcinogenic risk for children via inhalation was 2.64, with the non-carcinogenic risk of Pb for children comprising 1.96 of that risk. Compared to the results in three Silesian cities [32], the non-carcinogenic risks of Cd and Ni were much higher in Baoding than those found in Silesian. Hence, the total non-carcinogenic risk and the non-carcinogenic risk result from Pb for children should cause more public attention. The non-carcinogenic risks among the metals during the heating period showed the same trend as that observed during non-heating period. In comparison with the non-heating period, the non-carcinogenic risks of heavy metals to different populations increased. The non-carcinogenic risk result from Pb for children was 3.68 and the total heavy metal-related non-carcinogenic risk for children was 4.47. Because both values were greater than 1, policymaker and residents should pay more attention to the total and Pb-related non-carcinogenic inhalation risks to children. To sum up, the non-carcinogenic risks of all heavy metals through inhalation exposure pathway to children cannot be neglected and more serious attention should be paid to the non-carcinogenic risk caused by Pb.

##### Non-Carcinogenic Risks through the Ingestion Exposure Pathway

The non-carcinogenic risks of all heavy metals via ingestion during the whole sampling period are depicted in Table 9. During the non-heating period, the non-carcinogenic risks for all heavy metals were highest to children, followed by adult females and then adult males. Across the heavy metals studied, the non-carcinogenic risks descended following the order Cr > Cd > Pb > Cu > Zn > Ni, with a total non-carcinogenic risk for heavy metals to children of 1.20, indicating that the heave metal-related non-carcinogenic risks to children via ingestion could not be neglected. During the heating period, the trends were the same as those observed during the non-heating period. The non-carcinogenic risks for heavy metals to different populations were higher during the heating. During the heating period, the non-carcinogenic risk to children caused by Cr and the total non-carcinogenic risks of heavy metals were 1.13 and 1.47, respectively. Because both were greater than 1, these non-carcinogenic ingestion risks to children were deemed significant and corresponding measures should be taken to reduce potential harm.

By combining the results from Section 2.3.1, it appears that the non-carcinogenic risks for heavy metals were the highest via inhalation exposure, followed by ingestion exposure and finally dermal exposure. These three exposure pathways produced the greatest non-carcinogenic risks for children, and the non-carcinogenic risk of each heavy metal was higher during the heating period relative to the non-heating period. The non-carcinogenic risk caused by Pb via inhalation exposure and the non-carcinogenic risk of Cr through ingestion exposure were both extremely harmful to children, and should be paid more attention seriously.

#### 3.3.2. Carcinogenic Risk

##### Carcinogenic Risks through the Dermal Exposure Pathway

The carcinogenic risks of heavy metals through dermal exposure during the whole sampling period are depicted in Table 10. During the non-heating period, the carcinogenic risks of carcinogenic heavy metals were highest for adult females, followed by children and then adult males. The carcinogenic risk of Cr was greater than that of Ni across all three populations, and the total and individual metal carcinogenic risks ranged between 10^−4^ and 10^−6^, meaning that the carcinogenic risks from dermal exposure were acceptable. The carcinogenic risks of heavy metals were higher during heating period in comparison with the non-heating period, but the risk trends across populations and between metals stayed the same and were within the acceptable ranges.

##### Carcinogenic Risks through the Inhalation Exposure Pathway

The carcinogenic risks of each heavy metal to different populations through inhalation are depicted in Table 11. During the non-heating period, the carcinogenic risk to children via inhalation exposure was the highest, followed by adult males and then adult females, with the risk on a per-metal basis decreasing from Cr to Cd to Ni. The total carcinogenic risks to all populations were greater than 10^−4^, as well as the Cr-specific carcinogenic risks, indicating that all kind populations face significant carcinogenic risks due to heavy metals in general and Cr alone.

During the heating period, the trends across the total and metal-specific carcinogenic risks were the same as those observed during the non-heating period. The carcinogenic risks of heavy metals increased during the non-heating period and the total carcinogenic risks to different populations exceeded the thresholds, with the Cr-specific carcinogenic risks being the most significant. In summary, the Cr-specific carcinogenic risks from inhalation posed the highest risk to all populations, and should be paid more attention seriously.

##### Carcinogenic Risks through the Ingestion Exposure Pathway

The carcinogenic risks of each heavy metal from ingestion exposure to different populations are depicted in Table 12. The carcinogenic risks of heavy metals were the highest for adult females, followed by children and then adult males. Cr-specific carcinogenic risks were greater than Ni-specific carcinogenic risks for all populations. The metal-specific carcinogenic risks the total carcinogenic risks to different populations via ingestion exposure were all within the safe threshold range. The metal-specific carcinogenic risks to different populations increased during the heating period relative to the non-heating period, but the general trends across metals and populations were the same. The metal-specific and total metal carcinogenic risks to different populations through ingestion were all within the safe threshold range.

Combining the results from Section 2.3.2, it appeared that inhalation exposure caused the highest carcinogenic risk, followed by ingestion exposure and then dermal exposure. The carcinogenic risks to all populations via inhalation all exceeded the threshold range, and the Cr-specific carcinogenic risks contributed the most to the total carcinogenic risks through all pathways for all populations, and must be paid more attention seriously.

Combining the results from Section 2.3.1 and Section 2.3.2, it could be concluded that the risks to all populations were highest for inhalation exposure, followed by ingestion exposure and then dermal exposure, and that the risks to different populations were greater during the heating period in comparison with the non-heating period.

## 4. Conclusions

The average daily PM_2.5_ concentrations of the national air monitoring stations was 155.66 μg·m^−3^ which was 2.08 times as high as that of the second level criterion in China (75 μg·m^−3^) during the whole sampling period. All PM_2.5_-bound metals from Baoding were high, with Cd concentrations being consistently higher than the other measured metals. All heavy metals concentrations were higher during the heating period in comparison with the non-heating period. And the growth rates of Pb and Ni were the highest and the lowest, which were 88.03 and 5.11 percent, respectively.

The *EF* values for all heavy metals were higher during the heating period compared with the non-heating period, but there were no differences in the degree of each heavy metal. The *EF* values for Cr and Ni belonged to Grade 2, meaning that Cr and Ni were mildly enriched and affected by natural sources and human sources together. The *EF* values for Pb, Cu and Zn belonged to Grade 4 and thus significantly enriched, and the *EF* value of Cd was Grade 5 and thus extremely highly enriched. All four were mainly influenced by human factors. According to the pollution load index method, the pollution was more pronounced during the heating period in comparison with the non-heating period. The number of highly and very highly polluted PM_2.5_-bound metals was highest during the heating period, while the number of moderately pollution of PM_2.5_-bound metals was highest during the non-heating period. The combined pollution degree of heavy metal pollution was much pronounced during the heating period than the non-heating period.

According to the health risk assessment method proposed by U.S. EPA, the health risks to different populations in descending order were due to inhalation exposure, followed by ingestion exposure and finally dermal exposure, with the health risks being higher during the heating period. The non-carcinogenic risks for each heavy metal to children mainly resulted from inhalation and ingestion exposure, and the non-carcinogenic risks for Pb and Cr were greater to various kinds of people, and all the above non-carcinogenic risks exceeded the standard limits suggested by U.S. EPA (HI or HQ < 1), which should be taken seriously. During the whole sampling period, the carcinogenic risks to all populations through inhalation exposure exceeded the threshold ranges (10^−6^~10^−4^), and the carcinogenic risk caused by Cr was the greatest. Therefore, the carcinogenic and non-carcinogenic risks to all population age groups from PM_2.5_-bound metals concentrations must be considered. The results of health risks provided more useful information to the policymaker for atmospheric pollution control. And we also hope these results will be useful for residents to take protective measures to protect local environment.

## Figures and Tables

**Table 1:** The standard of enrichment factor.

Level	*EF*	Degree of Enrichment	Source
First	<1	No enrichment	Crust and soil
Second	1~10	Minimal enrichment	Natural factors and human factors
Third	10~100	Moderate enrichment	Human factors
Fourth	100~1000	Significant enrichment	Human factors
Fifth	>1000	Extremely high enrichment	Human factors

**Table 2:** The standard of pollution load index.

Level	*PLI*	Pollution Level
First	=0	Background concentration
Second	1~2	Unpolluted
Third	2~3	Mildly polluted
Fourth	3~4	Moderately polluted
Fifth	4~5	Highly polluted
Sixth	>5	Very highly polluted

**Table 3:** The exposure parameter values.

Parameter	Physical Significance	Value			Unit
		Children	Adult Males	Adult Females	
SA	Exposed skin area	2800	3300	3300	cm^2^·d^−1^
AF	Skin adherence factor	0.2	0.2	0.2	mg·cm^−2^
ABS	Dimensionless dermal absorption factor	-	-	-	-
EF	Exposure relative frequency	365	365	365	d·a^−1^
ED	Exposure duration	18	30	30	a
CF	Conversion coefficient	10^−6^	10^−6^	10^−6^	kg·mg^−1^
IR_i_	Inhalation rate	8.7	15.2	11.3	m^3^·d^−1^
BW	Average body rate	44	62.7	54.4	kg
IngR	Ingestion rate	250	150	150	mg·kg^−1^
AT	Averaging time (non-carcinogens)	2190	10,950	10,950	d
	Averaging time (carcinogens)	25,550	25,550	25,550	d

Notes: The dimensionless dermal absorption factor (ABS) is 0.01 for all elements except Cd, for which ABS is 0.001.

**Table 4:** Reference doses of heavy metals through three exposure pathways (mg·(kg·d)^−1^).

Element	*RfD* via Dermal Exposure	*RfD* via Inhalation Exposure	*RfD* via Ingestion Exposure
Cr	6.00 × 10^−5^	2.86 × 10^−5^	3.00 × 10^−3^
Pb	5.25 × 10^−4^	3.52 × 10^−3^	3.50 × 10^−3^
Ni	5.40 × 10^−3^	2.06 × 10^−2^	2.00 × 10^−2^
Cd	1.00 × 10^−5^	1.00 × 10^−3^	1.00 × 10^−3^
Cu	1.20 × 10^−2^	4.02 × 10^−2^	4.00 × 10^−2^
Zn	6.00 × 10^−2^	3.00 × 10^−1^	3.00 × 10^−1^

**Table 5:** Carcinogenic factors of heavy metals through three exposure pathways (mg·(kg·d)^−1^).

Element	*SF* via Dermal Exposure	*SF* via Inhalation Exposure	*SF* via Ingestion Exposure
Cr	2.00 × 10	4.20 × 10	5.00 × 10^−1^
Ni	4.25 × 10	8.40 × 10^−1^	8.40 × 10^−1^
Cd	—	6.40	—

**Table 6:** Comparison of PM_2.5_-bound metals concentrations from different cities (ng·m^−3^).

Cities	Cr	Pb	Ni	Cd	Cu	Zn
Baoding (overall period)	49.59 ± 17.14	564.09 ± 411.39	12.21 ± 5.94	19.52 ± 11.23	186.18 ± 170.32	489.67 ± 297.21
Baoding (non-heating period)	45.04 ± 15.35	402.11 ± 341.38	11.93 ± 3.31	18.83 ± 13.16	165.41 ± 150.47	441.23 ± 236.12
Baoding (heating period)	54.98 ± 17.86	756.07 ± 409.90	12.54 ± 5.57	20.34 ± 8.57	210.80 ± 191.19	547.08 ± 352.52
Beijing	11.30 ± 9.40	142.50 ± 98.90	4.0 ± 2.40	2.60 ± 2.40	—	—
Tianjin	23.0 ± 8.35	101.0 ± 46.71	—	1.0 ± 0..69	68.0 ± 49.72	1144.0 ± 685.69
Chengdu	17.9 ± 10.5	320.50 ± 186.0	5.1 ± 4.10	5.90 ± 10.70	—	—
Shanghai	19.0 ± 17.0	75.0 ± 74.0	6.0 ± 4.0	1.70 ± 1.60	33.0 ± 20.0	344.0 ± 274.0
Guangzhou	70.0 ± 20.0	450.0 ± 210.0	—	20.0 ± 10.0	190.0 ± 80.0	1360.0 ± 500.0
Chongqing	190.0 ± 100.0	320.0 ± 120.0	30.0 ± 30.0	70.0 ± 40.0	60.0 ± 20.0	600.0 ± 280.0
Xiamen	22.0 ± 9.0	58.1 ± 29.0	1.90 ± 0.90	1.80 ± 0.50	48.5 ± 21.40	138.70 ± 43.30

“—” represents no data.

**Table 7:** The non-carcinogenic risks of heavy metals via the dermal exposure pathway.

Elements	Non-Heating Period			Heating Period		
	Children	Adult Males	Adult Females	Children	Adult Males	Adult Females
Cr	5.73 × 10^−3^	1.58 × 10^−3^	1.82 × 10^−3^	7.00 × 10^−3^	1.93 × 10^−3^	2.22 × 10^−3^
Pb	1.10 × 10^−1^	3.02 × 10^−2^	3.48 × 10^−2^	2.06 × 10^−1^	5.68 × 10^−2^	6.55 × 10^−2^
Ni	8.43 × 10^−4^	2.33 × 10^−4^	2.68 × 10^−4^	8.86 × 10^−4^	2.44 × 10^−4^	2.82 × 10^−4^
Cd	2.87 × 10^−2^	7.91 × 10^−3^	9.12 × 10^−3^	3.10 × 10^−2^	8.54 × 10^−3^	9.85 × 10^−3^
Cu	5.26 × 10^−3^	1.45 × 10^−3^	1.67 × 10^−3^	6.71 × 10^−3^	1.85 × 10^−3^	2.13 × 10^−3^
Zn	2.81 × 10^−3^	7.74 × 10^−4^	8.92 × 10^−4^	3.48 × 10^−3^	9.60 × 10^−4^	1.11 × 10^−3^
Sum	1.53 × 10^−1^	4.22 × 10^−2^	4.86 × 10^−2^	2.55 × 10^−1^	7.04 × 10^−2^	8.11 × 10^−2^

**Table 8:** The non-carcinogenic risks of heavy metals via inhalation.

Elements	Non-Heating Period			Heating Period		
	Children	Adult Males	Ault Females	Children	Adult Males	Ault Females
Cr	2.56 × 10^−1^	4.14 × 10^−2^	3.59 × 10^−2^	3.12 × 10^−1^	5.05 × 10^−2^	4.38 × 10^−2^
Pb	1.96	3.17 × 10^−1^	2.75 × 10^−1^	3.68	5.96 × 10^−1^	5.17 × 10^−1^
Ni	1.02 × 10^−2^	1.64 × 10^−3^	1.43 × 10^−3^	1.07 × 10^−2^	1.73 × 10^−3^	1.50 × 10^−3^
Cd	3.21 × 10^−1^	5.19 × 10^−2^	4.51 × 10^−2^	3.47 × 10^−1^	5.61 × 10^−2^	4.87 × 10^−2^
Cu	7.05 × 10^−2^	1.14 × 10^−2^	9.89 × 10^−3^	8.98 × 10^−2^	1.45 × 10^−2^	1.26 × 10^−2^
Zn	2.51 × 10^−2^	4.06 × 10^−3^	3.52 × 10^−3^	3.11 × 10^−2^	5.03 × 10^−3^	4.36 × 10^−3^
Sum	2.64	4.27 × 10^−1^	3.71 × 10^−1^	4.47	7.24 × 10^−1^	6.28 × 10^−1^

**Table 9:** The non-carcinogenic risks of heavy metals via ingestion.

Elements	Non-Heating Period			Heating Period		
	Children	Adult Males	Ault Females	Children	Adult Males	Ault Females
Cr	9.34 × 10^−1^	2.27 × 10^−1^	2.52 × 10^−1^	1.13	3.95 × 10^−1^	4.62 × 10^−1^
Pb	6.82 × 10^−2^	2.39 × 10^−2^	2.79 × 10^−2^	1.23 × 10^−1^	4.32 × 10^−2^	5.04 × 10^−2^
Ni	3.43 × 10^−4^	1.20 × 10^−4^	1.40 × 10^−4^	3.63 × 10^−4^	1.27 × 10^−4^	1.48 × 10^−4^
Cd	1.96 × 10^−1^	6.85 × 10^−2^	8.00 × 10^−2^	2.12 × 10^−1^	7.43 × 10^−2^	8.67 × 10^−2^
Cu	2.45 × 10^−3^	8.59 × 10^−4^	1.00 × 10^−3^	3.10 × 10^−3^	1.08 × 10^−3^	1.27 × 10^−3^
Zn	8.72 × 10^−4^	3.06 × 10^−4^	3.57 × 10^−4^	1.01 × 10^−3^	3.55 × 10^−4^	4.15 × 10^−4^
Sum	1.20	3.21 × 10^−1^	3.61 × 10^−1^	1.47	5.14 × 10^−1^	6.00 × 10^−1^

**Table 10:** The carcinogenic risks of heavy metals via dermal exposure.

Elements	Non-Heating Period			Heating Period		
	Children	Adult Males	Ault Females	Children	Adult Males	Ault Females
Cr (VI)	1.66 × 10^−5^	2.29 × 10^−5^	2.64 × 10^−5^	1.74 × 10^−5^	2.40 × 10^−5^	2.77 × 10^−5^
Ni	7.37 × 10^−7^	1.02 × 10^−6^	1.17 × 10^−6^	9.00 × 10^−7^	1.24 × 10^−6^	1.43 × 10^−6^
Sum	1.73 × 10^−5^	2.39 × 10^−5^	2.75 × 10^−5^	1.83 × 10^−5^	2.53 × 10^−5^	2.91 × 10^−5^

**Table 11:** The carcinogenic risks of heavy metals via inhalation.

Elements	Non-Heating Period			Heating Period		
	Children	Adult Males	Ault Females	Children	Adult Males	Ault Females
Cr (VI)	1.97 × 10^−4^	1.68 × 10^−4^	9.62 × 10^−5^	2.38 × 10^−4^	2.04 × 10^−4^	1.16 × 10^−4^
Ni	1.05 × 10^−6^	8.94 × 10^−7^	5.27 × 10^−7^	1.10 × 10^−6^	9.43 × 10^−7^	5.40 × 10^−7^
Cd	1.25 × 10^−5^	1.07 × 10^−5^	6.13 × 10^−6^	1.36 × 10^−5^	1.16 × 10^−5^	6.64 × 10^−6^
Sum	2.10 × 10^−4^	1.80 × 10^−4^	1.03 × 10^−4^	2.52 × 10^−4^	2.16 × 10^−4^	1.23 × 10^−4^

**Table 12:** The carcinogenic risks of heavy metals via ingestion.

Elements	Non-Heating Period			Heating Period		
	Children	Adult Males	Ault Females	Children	Adult Males	Ault Females
Cr (VI)	2.66 × 10^−5^	2.31 × 10^−5^	3.29 × 10^−5^	3.25 × 10^−5^	2.82 × 10^−5^	4.02 × 10^−5^
Ni	1.18 × 10^−5^	1.03 × 10^−5^	1.46 × 10^−5^	1.24 × 10^−5^	1.08 × 10^−5^	1.54 × 10^−5^
Sum	3.85 × 10^−5^	3.34 × 10^−5^	4.75 × 10^−5^	4.49 × 10^−5^	3.89 × 10^−5^	5.56 × 10^−5^
